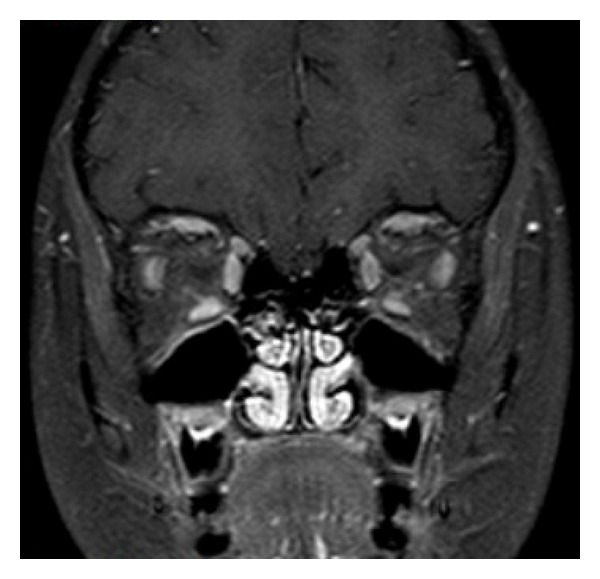# Myositic Type of Idiopathic Orbital Pseudotumor in a 4-Year-Old Child: A Case Report

**DOI:** 10.1155/2012/473856

**Published:** 2012-06-10

**Authors:** Selim Bakan, Ayse Ahsen Bakan, Abdullah Kursat Cingu, Mehtap Beker Acay, Ahmet Gocmez, Ahmet Elbeyli, Serra Sencer

**Affiliations:** ^1^Department of Radiology, Viransehir State Hospital, 63100 Sanliurfa, Turkey; ^2^Department of Ophthalmology, Dicle University School of Medicine, 21080 Diyarbakir, Turkey; ^3^Department of Radiology, Corum State Hospital, 19000 Corum, Turkey; ^4^Department of Radiology, Istanbul University School of Medicine, 34452 Istanbul, Turkey; ^5^Department of Ophthalmology, Viransehir State Hospital, 63100 Sanliurfa, Turkey; ^6^Department of Neuroradiology, Istanbul University School of Medicine, 34452 Istanbul, Turkey

## Abstract

Idiopathic orbital pseudotumor is a benign, noninfectious, and nonneoplastic disease with unknown cause. It is the third most common orbital disease after thyroid orbitopathy and lymphoproliferative disorder. Idiopathic orbital pseudotumor is extremely rare in pediatric age group and may cause real diagnostic problems. This paper describes a 4-year-old girl who presented with sudden ptosis in the right eye and swollen eyelid. She recovered completely with high-dose steroid therapy. We report clinical and magnetic resonance imaging findings of orbital myositis, which is a rare subtype of idiopathic orbital pseudotumor in children and needs to be differentiated from other orbital disease especially malignancy.

## 1. Introduction

Idiopathic orbital pseudotumor (IOP) is a nongranulomatous inflammatory process in the orbit with no known local or systemic causes. After Graves' disease and lymphoproliferative disorders, orbital pseudotumor is the third most common ophthalmologic disease of the orbit and accounts for approximately 8–11% of all orbital tumors. The most frequent subtype of IOP is a local mass within the orbit (50%), followed by dacryoadenitis (29%), myositis (8%), perineuritis (4%), eyelid pseudotumor (4%), and diffuse orbital inflammation (4%) [1]. Pediatric cases account for 11.5% of the total population of cases with IOP [2], and several conditions such as cellulites, rhabdomyosarcoma, and leukemia are considered in the differential diagnosis in children [3]. Magnetic resonance imaging (MRI) plays an important role in imaging diagnosis [4]. In this case report, we present an unusual case of myositic type of IOP in a child.

## 2. Case Presentation

**Figure 1:**
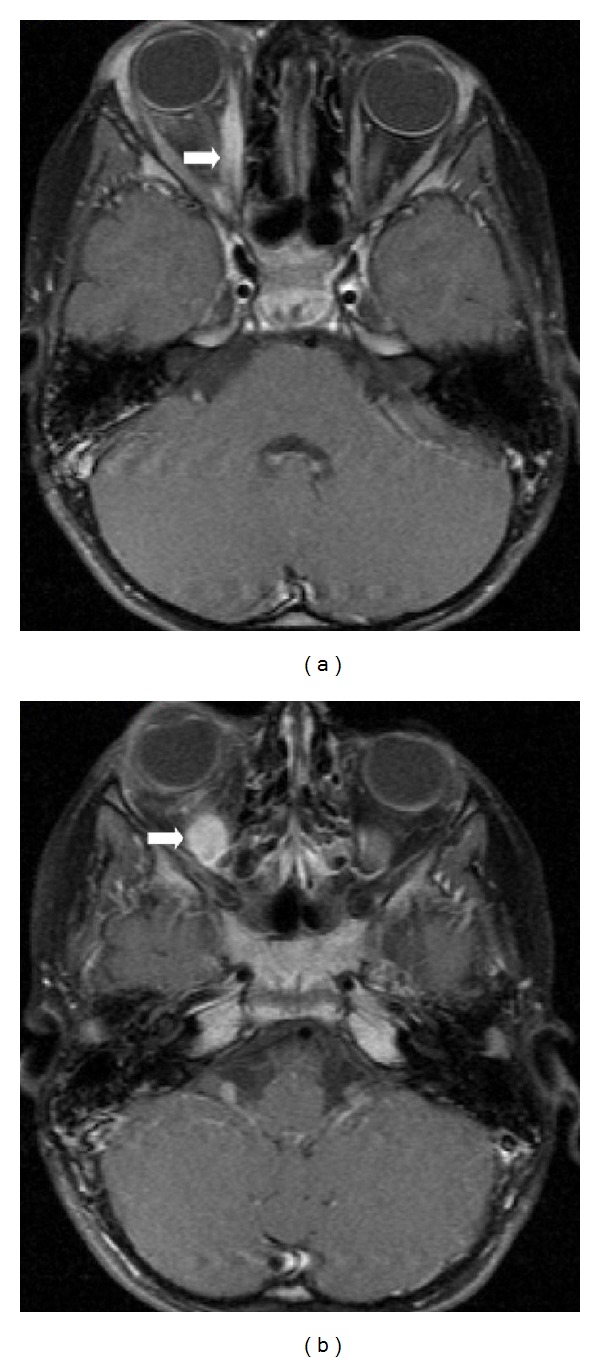


**Figure 2:**
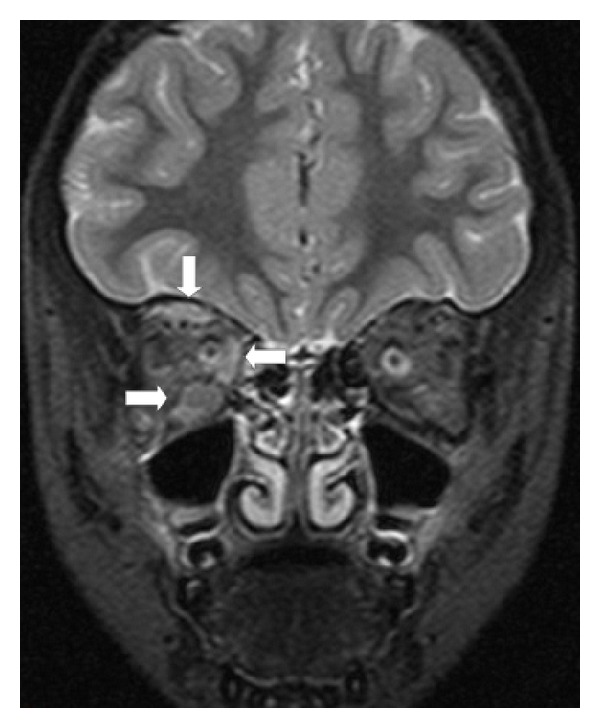


A previously healthy and normally developing 4-year-old girl presented to our pediatric clinic with a 2-day history of painless, gradually progressing ptosis and swollen right eyelid. Visual acuity was 20/20 in her both eyes. Ophthalmological examination of the patient was normal other than upper eyelid ptosis, proptosis, and ophthalmoplegia in the right eye. She was not able to use her left leg and left arm at admission. For this reason, cranial computed tomography (CT) was performed to check for intracranial hemorrhage or mass and turned out to be normal. Complete blood count, electrolyte levels, and sedimentation rate were in normal ranges. There was no atypical cell in peripheral blood smear. In orbital MRI examination, postgadolinium fat-saturated T1-weighted images revealed marked contrast enhancement of medial (Figure 1(a)) and inferior rectus (Figure 1(b)) muscles. MRI also showed enlargement of superior, inferior, and medial rectus muscles and their tendons in the right eye with minimally hyperintensity on fat-saturated T2-weighted image (Figure 2). A preliminary diagnosis of IOP was made, although it is rare in a child. The child was decided to be treated with intravenous high-dose steroids before considering a biopsy. She began to show signs of clinical improvement, including reduced proptosis and ptosis within 36 hours after starting high-dose steroid treatment. She continued with intravenous high-dose steroids for 3 full days before she was discharged on a gradually tapering dose of oral prednisone. Two weeks after initial examination, follow-up orbital MRI demonstrated a reduction in the size and contrast enhancement of effected muscles in postgadolinium fat-saturated T1-weighted images (Figure 3).

## 3. Discussion

First described by Birch-Hirschfield in 1905, idiopathic orbital inflammatory syndrome, also known as IOP, is a nonspecific, nongranulomatous inflammatory process of the orbit with spontaneous resolution [2]. Orbital myositis is a common component of IOP. Although IOP is bilateral in 45% of pediatric cases, in general 90–95% of the cases are unilateral as seen in our case [3, 5]. In 50% of the child patients, systemic signs of IOP may include headache, emesis, anorexia, lethargy, and fever, although these symptoms are rarely reported in adult patients [3, 6]. Ocular motility restriction, swollen eyelid, proptosis, and high orbital pressure are the most common presenting signs in children with IOP. Ptosis occurred more often in pediatric cases than in adult cases [2]. Routine laboratory investigations obtained during the evaluation of IOP are typically normal with the exception of an elevated leukocyte count, sedimentation rate, and eosinophilia [7]. Although these features suggest an active inflammatory process, they are not specific for IOP. A high degree of clinical suspicion combined with neuroimaging results is more helpful in making a correct diagnosis.

Radiologic findings in IOP are characterized by inflammatory changes in various intraorbital structures, such as the globe, lachrymal glands, extraocular muscles, orbital fat, and optic nerve [8]. MRI is reported to be superior to CT, which should be avoided if possible due to the risk of radiation, especially in children, in detecting extraorbital or intracranial extensions in this disorder [9]. Although on conventional MRI sequences, subtle areas of inflammation or enhancing tissues can easily be masked by the high-signal intensity of orbital fat, and involvement of the fat itself may not be appreciated. Thus, it is very important to use frequency-selective fat saturation and contrast-enhanced sequences for patients with IOP [4].

Some of the differential diagnosis of pediatric IOP may include orbital cellulites, rhabdomyosarcoma, leukemia, orbital trauma with retained foreign body, ruptured dermoid cyst, lymphangioma, neuroblastoma, metastatic retinoblastoma, and thyroid related orbitopathy [3]. Enlargement of only a single extraocular muscle or a combination of several muscles occur frequently in IOP. The tendons enlarge together with the muscle bundles and lead to a tubular configuration. This muscle involvement is in contrast to thyroid ophthalmopathy, in which all muscles are effected and have a reveal a spindle-shaped configuration with normal tendons [10]. The common cause of orbital cellulitis is sinusitis, and IOP is differentiated from cellulitis by the absence of contiguous paranasal sinus process [11]. A dermoid or epidermoid cyst is depicted on CT or MRI as a well-circumscribed cystic lesion that may contain fat or foci of calcification and fat-fluid levels and may have slight enhancement of its wall [12].

IOP can mimic malignant tumors both clinically and radiologically. Both IOP and lymphoid tumors appeared isointense to muscles on T1-weighted images. Patients with IOP show isointense and lymphoid tumors hyperintense to fat on T2-weighted images. Also IOP patients reveal enhancement with contrast more prominent than observed in lymphoma cases [10, 13].

Systemic corticosteroid therapy is the cornerstone of managing IOP. Similar to our case, over 75% of patients show dramatic improvement within 24 to 48 hours of treatment [14]. Improvement with corticosteroid therapy is of diagnostic significance, and specifically a corticosteroid responsive orbital process is more likely to correspond to pseudotumor [15].

## 4. Conclusion

In conclusion, IOP is not common in pediatric age group, and extraocular muscle involvement by itself is rare. Awareness of MR imaging features of IOP provides a better differential diagnosis and prevents unnecessary biopsies. Follow-up MRI examination, performed shortly after the initiation of steroid treatment, is necessary in order to reveal the improvement of the findings and confirms the suggested diagnosis.

## Figures and Tables

**Figure 3:**